# Exploration on the Application Path of College English MOOCS Teaching under the Background of Internet of Things

**DOI:** 10.1155/2022/4572432

**Published:** 2022-05-21

**Authors:** Daoping Xu

**Affiliations:** School of International Business, Qingdao Huanghai University, Qingdao, Shandong 266427, China

## Abstract

While the development of information and communication technology has brought great changes to social industrial structure and economic development, it has also reshaped people's cognition and thinking modes to a great extent and has paid attention to the field of education under the guidance of technology. The reform of education is taking place. According to the background of the Internet of things, the teaching quality evaluation index is reconstructed with the help of principal component analysis, support vector machine, and fuzzy algorithms, and the application performance and practical results of the improved teaching path are analyzed. The results show that the evaluation accuracy of the improved hybrid algorithm for teaching quality reaches more than 90%, and its evaluation time is far less than that of the SVM algorithm (10.23 s), and it has a good application effect on the evaluation of teaching quality. The increase of students' satisfaction with the improved teaching quality has reached 5.8%, and their professional skills have improved by 6–25 points. After expert evaluation, it is found that the maximum satisfaction frequency of teaching content and teaching effect can reach 96.7% and 91.3%, respectively. Reconstructing the index system of the English path under the background of the Internet of things can better grasp students' learning needs and integrate multiple methods to provide new research ideas for follow-up English teaching.

## 1. Introduction

Information-based teaching resources and teaching achievements are widely used in education and teaching. As a basic course of higher education teaching, English should always adhere to the principle of innovative teaching in teaching. Since 2018, education informatization 2.0, published by the ministry of education, proposed that innovative research on intelligent education should be actively carried out by using emerging technologies, intelligent devices, and networks, integrate information technology into College English teaching and explore distance teaching from a new perspective. The teaching method of massive open online courses (MOOC/MOOCS) has greatly improved the “individualization” and “personalization” in the teaching process. The innovative teaching method has enriched the teaching activities and mobilized the active participation of students. However, the teaching activity mainly depends on online resources, and the binding force on students is mainly through the compulsory completion of course homework. It cannot be shown that it will make students complete their homework in order to complete the course, which will have a certain impact on students' learning consciousness and the self-binding force of the course. This research reconstructs the teaching path by integrating a variety of algorithms to explore its application performance, which can effectively ensure the completion of its teaching objectives and the improvement of students' professional quality.

By introducing principal component analysis (PCA) and integrating it with a support vector machine, the problem of teaching quality is transformed into a classification problem. The principal component analysis is to extract a set of nonlinear main variable relationships from possibly related data through orthogonal change; that is, to realize data dimensionality reduction for factor analysis. At the same time, the network analysis method is used to reconstruct the indicators affecting the teaching path, and the expert evaluation method is used to score the satisfaction of the index system, so as to make the improvement of the English MOOCS teaching path more convincing and provide guarantee for the follow-up students' learning quality.

The innovation of this research lies in the combination of principal component analysis and support vector machine to construct the teaching quality index system and adding fuzzy algorithm and expert evaluation to quantitatively reconstruct and analyze the indicators, so as to make the establishment of teaching quality and path more objective and comprehensive, more in line with the teaching concept, and jump out of the subjectivity of the traditional single evaluation method [1].

This paper mainly studies the innovation of the College English MOOCS teaching path under the Internet of things from four aspects. The second part comprehensively discusses some research progress and methods on MOOCS teaching and English teaching methods [2]. The third part introduces principal component analysis and support vector machine, establishes the index system model, optimizes and evaluates the index system with a fuzzy algorithm and expert review, and tests the application performance of the model [3]. The fourth part evaluates the application performance and practical effect of the model, and arranges and analyzes the results [4]. The last part is the summary of the whole article [5].

## 2. Related Works

Konstantinidis et al. pointed out the role of Internet 0 of things technology in education, summarized the educational concept under the Internet of things, and discussed its future development prospect [6]. In order to better ensure the comfort of learning, Marques et al. put forward an Internet of things solution for noncontact measurement and real-time temperature monitoring, which makes an important contribution to the infrared temperature monitoring system of the Internet of things [7]. Starting from the actual situation of College English teaching, Zhang et al. discuss its countermeasures from the perspective of cultural differences, so as to provide a valuable reference for English teaching [8, 9]. Li et al. have determined the characteristics, platforms, course materials, and methods to promote interaction adopted by MOOC through the teaching situation and relevant cases of previous campus courses, and provided methods for students to provide flexible and personalized campus course learning experiences [10]. Holme et al. analyze whether these resources meet the actual needs of English teachers by investigating and comparing teachers' use of information technology [11]. Hong et al. have changed the difficulties of traditional teaching methods in students' learning of microbiology and adopted a variety of modern multimedia teaching methods to ensure the teaching quality [12] and proposed the collaborative role of computer support and encouraged teachers to actively integrate MOOCs into the improvement of the teaching path [13].

Gao et al. pointed out that the cramming teaching mode of traditional English makes students lack enthusiasm and initiative and advocated the introduction of the MOOC teaching mode to teach students' reading ability and expression ability [14]. Pan et al. study the innovation of MOOC curriculum development and teaching methods, and their opinions on teaching methods and theoretical models help to develop soft skills in MOOC [15]. Hao et al. found that curriculum design is of great significance for MOOC teaching platforms through questionnaires and software simulations, and encouraged the use of information technology tools in the context of distance education and online learning [16]. Dehua et al. proposed that the way of teaching and learning should be handled according to its actual situation [17]. Hilles et al. established a mathematical model of oral English teaching with metacognition and found that the model has a good operational effect in its follow-up simulation test [18]. Corpuz et al. introduce the feasibility of designing a text classification model using two popular and robust algorithms: support vector machine (SVM) and long-term and short-term memory (LSTM), which are used to automatically classify complaints, suggestions, feedback, and praise. The degree of preference between the two algorithms can be attributed to the available data sets and the skills of optimizing these algorithms through feature engineering technology and applying them to practical text classification applications [13]. Hong et al. provided customer evaluation data by analyzing the correlation between airline customer satisfaction based on customer evaluation data. [19]. Mikalef et al. and Wilson introduced fuzzy mathematics and machine learning algorithm to evaluate the application of education and teaching quality model through the functional relationship between teaching quality evaluation index and teaching effect and found that machine learning algorithm has good reliability in evaluation [20, 21]. The pan teams proposed algorithm extraction and emergency division based on English feature rules to predict the control reality established by the algorithm, which has a good application effect [22].

To sum up, it can be found that most researchers begin to pay attention to the improvement of teaching path and teaching quality, integrate computer, and machine algorithms into MOOCS teaching methods, and actively introduce metacognition, support vector machine, and neural network model analysis, but rarely integrate and explore a variety of algorithms [23]. Therefore, by proposing the teaching path exploration integrating multiple algorithms, students can effectively learn with the help of information technology tools and improve their professional quality [24].

## 3. Research on the Application Path and Model Construction of College English MOOCS Teaching under the Background of Internet of Things

### 3.1. Reform and Exploration of English Teaching Path under the Background of the Internet of Things

**Figure 1:**
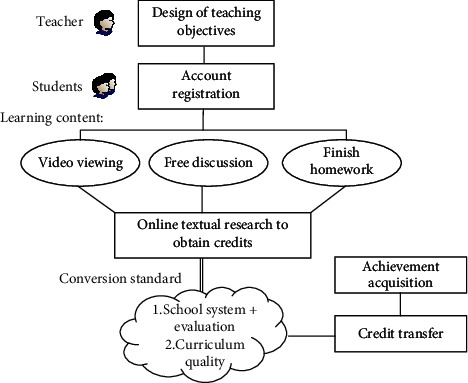
Available MOOCS teaching mode diagram.

Smart classroom integrates online and offline courses, combines mixed teaching and flipped classroom, and realizes the full monitoring of students' learning process with the help of Internet technology. Build a college English smart classroom teaching system, design the close connection of various teaching links, realize a series of smart teaching activities such as intelligent resource push and diversified teaching rating, and truly realize the comprehensive personality of College English classroom teaching. Starting from two subjects, students and teachers, this study uses the principal component analysis method to reconstruct the original teaching quality index. Select some data to form training samples, and analyze and calculate the application performance impact of the innovation path according to the weight ratio affecting teaching quality. The construction of its teaching path and mechanism is shown in Figure 1.

MOOC, as a new teaching mode with the development of information technology, is different from the traditional teaching mode in many aspects. The traditional teaching model includes stimulating students' learning motivation, reviewing old knowledge, teaching new knowledge, consolidating exercises and homework. Although traditional teaching also emphasizes the systematicness of knowledge and the two-way nature of teaching, teachers occupy an absolute leading position in teaching. Students only need to cooperate with teachers' teaching activities on the basis of maintaining their initiative. Under MOOC, learners need to choose learning contents, methods, and progress according to their own needs and future development needs and respect the individual differences of students' development. Based on the mastery learning theory, it is believed that learners do not have great differences in learning ability and learning motivation. As long as they study carefully, they can master the learning content.

MOOCS curriculum model re-establishes students' learning methods with the help of online learning resources, breaks through the traditional face-to-face teaching and online teaching methods and has become a more popular teaching method with the advantages of diversified resources and tools, wide audience, and high independent participation. At the same time, the MOOCS course is mandatory to complete a homework assignment, which can ensure the integrity of students' learning process. The main advantage of this model is to obtain higher income with less investment. This teaching method integrates online learning and resource integration and sharing, making teaching resources available and giving learners greater autonomy and creativity. Hierarchical learning progress and rich means of knowledge acquisition can achieve better learning results.

### 3.2. Construction of English Teaching Model Indicators Based on Principal Component Analysis

The MOOCS teaching mode lacks an overall tracking and supervision mechanism in learning situation analysis, teaching design, and teaching implementation. According to the actual situation of students completing their homework on time, it is difficult to evaluate the overall state of students. The lack of diversity of teaching activities and the process of teaching implementation make there few interaction channels between teachers and students. The index system of teaching quality and teaching path is established by introducing principal component analysis; that is, the weight of each principal component is assigned, which not only reduces the workload but also reduces the interference of human factors in the evaluation process. Its mathematical expression is as follows:

(1) $$\begin{array}{c} xij^{*}=\frac{\left(xij-\bar{x}j\right)}{\sqrt{\sigma_{ii}}},\;i=1,2,\ldots ,n;\;j=1,2,\ldots ,p. \end{array}$$

Equation (1) is the standardized calculation formula, where *xij* is the original data of the *j* index of the *i* sample, *σii* is the average value of the *j* index original data of all samples, and *σii* is the variance of the *j* index original data of all samples, so as to obtain the standardized matrix, and calculate the correlation coefficient and variance contribution rate of the matrix to obtain the following equation:

(2) $$\begin{array}{c} \left\{\begin{array}{c} R=\left[rij\right]pxp=\frac{\left(Z^{T}Z\right)}{n-1},\\ \frac{\sum_{i=1}^{m}\lambda i}{\sum_{i=1}^{p}\lambda i}\geq 85\%. \end{array}\right. \end{array}$$

In equation (2), *R* is the correlation coefficient matrix, where *r*_*ij*_ is the correlation coefficient of the original variables *x*_*i*_ and *x*_*j*_, and *Z* is the standardization result of the original data matrix. The index system is reconstructed by judging the contribution rate of variance. If the contribution rate is greater than 85%, it shows that the index plays an important role in the evaluation of teaching quality, in which *λ*_*i*_ is the contribution rate of *i* main component and *m* is the number of main components. The evaluation destination of teaching quality is the classification problem. The establishment of support vector machine classifier can better improve the evaluation system of the index system, and its calculation formula can be expressed as the following formula:

(3) $$\begin{array}{c} \left\{\begin{array}{c} y1=\omega^{T}\phi \left(x\right)+b,\\ y2=\min J\left(\omega ,\xi \right)=\frac{1}{2}{\left\|\omega \right\|}^{2}+C\sum_{i=1}^{n}\xi i. \end{array}\right. \end{array}$$

Equation (3) is the hyperplane formula established by the support vector machine, where *x*_*i*_ is the evaluation index of English teaching quality, *y*_*i*_ is the grade of English teaching quality, *ω* is the normal vector of the hyperplane, *b* is the offset vector, and *C* is the penalty parameter, that is, the penalty degree of right and wrong samples. At the same time, when dealing with the classification problem of large samples, the classification problem of the support vector machine can be transformed into its dual problem by introducing the langrange multiplier, so as to speed up the running speed. The calculation formula of the hyperplane classification function is shown in the following formula:

(4) $$\begin{array}{c} f\left(x\right)=\text{sign}\left(\sum_{i=1}^{l}\alpha iyik\left(xi\cdot x\right)\right)+b. \end{array}$$

In equation (4), sign represents the symbolic function, *α*_*i*_ represents the langrange multiplier, and *K*(*x*_*i*_, *x*) represents the kernel function, that is, the point product. For the effect evaluation of teaching quality, it is difficult to be accurately analyzed quantitatively. The usual knowledge test is often used to establish the index system. Therefore, it is necessary to introduce the fuzzy mathematics method to convert the values to reduce the subjective fuzziness of experts in the evaluation. The calculation formula of triangular fuzzy numbers is shown in the following formula:

(5) $$\begin{array}{c} \mu_{A}\left(x\right)=\left\{\begin{array}{cc} \frac{x-m}{n-m}, & m<x<n;\\ \frac{x-r}{n-r}, & n<x<r;\\ 0, & \text{other.} \end{array}\right. \end{array}$$

*μ*_*A*_(*x*) is the expression of the membership function, where *U* is the final domain in the function, which is any fuzzy subset, *μ*_(*x*)_ ∈ [0,1] is the membership degree of the subset to the final domain, *μ* is the membership function, and the triangular fuzzy number *A* is (*m*, *n*, *r*), *μ*_*A*_(*x*) ranges between [0,1], *m*, *n*, *r* are all real numbers, which are the upper limit, most likely value and lower limit *r* of *A*, respectively. Arbitrary triangular fuzzy numbers *A*_1_(*m*_1_, *n*_1_, *r*_1_) and *A*_2_(*m*_2_, *n*_2_, *r*_2_) need to satisfy in the following formula:

(6) $$\begin{array}{c} \left\{\begin{array}{c} A_{1}+A_{2}=\left(m_{1}+m_{2},n_{1}+n_{2},r_{1}+r_{2}\right),\\ A_{1}-A_{2}=\left(m_{1}-m_{2},n_{1}-n_{2},r_{1}-r_{2}\right),\\ \lambda A_{1}=\left(\lambda m_{1},\lambda n_{1},\lambda r_{1}\right),\\ A_{1}⊗A_{2}=\left(m_{1}m_{2},n_{1}n_{2},r_{1}r_{2}\right). \end{array}\right. \end{array}$$

In order to reduce the subjective error of experts in the evaluation, it is necessary to carry out a triangular fuzzy transformation on the expert evaluation results to obtain the specific values that can be calculated, and finally, explain the fuzzy in detail. The application of the center of gravity method, which is less affected by the preference of decision-makers, can make the calculation relatively simple, and its expression formula is shown in the following formula:

(7) $$\begin{array}{c} f=\frac{\left(r-m\right)+\left(n-m\right)}{3}+m. \end{array}$$

In equation (7), *f* represents the clear value obtained by the center of gravity method.

### 3.3. Establishment of Teaching Innovation Mode under the Integration Path Model

**Figure 2:**
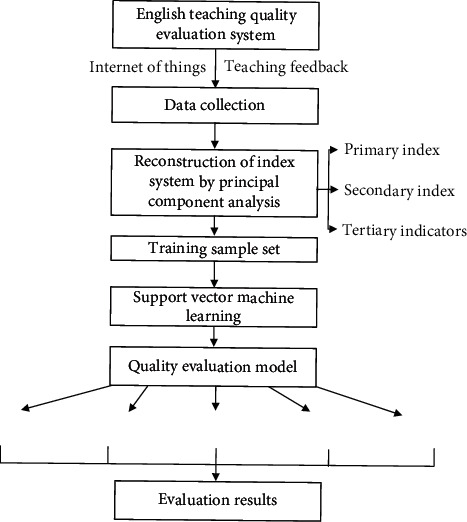
Structural diagram of teaching path innovation.

In the current College English teaching process, teachers mainly formulate teaching materials in the selection criteria of teaching materials and rarely involve the recommendation and guidance of expanded literature. In this teaching process, due to inevitable cultural differences and language habits, it is difficult for students to learn basic knowledge and improve their ability, and there are many standards for the evaluation of teaching quality. Some evaluation indicators deviate from student-centered teaching principle and the step-by-step teaching process. Through the introduction of the analytical network process (ANP), the influencing factors are analyzed, and the gray relationship application analysis is carried out on the scores of students after the change of innovation path. The structural diagram of teaching path innovation is shown in Figure 2.

The ANP method is an analysis method based on the analytic hierarchy process. It is applicable to the structural system under the interaction of elements. It has good flexibility in the process of practical application. Through the construction of the weighting matrix, the weighting matrix is obtained after standardizing the device, and the weight value of each influencing factor in the matrix is calculated. Set *T*_*d*_=[*t*_*ij*_^*D*^]_*m*×*m*_, *T*_*c*_=[*t*_*ij*_]_*n*×*n*_ as the primary and secondary index matrix, the standardization process is shown in the following equation:

(8) $$\begin{array}{c} T_{c}^{11}=\begin{array}{c} \ldots \\ c_{11}\\ \ldots \\ c_{1i}\\ \ldots \\ c_{1m_{1}} \end{array}\left[\begin{array}{ccccc} c_{11} & c_{1j} & \ldots & \ldots & c_{1m_{2}}\\ t_{11}^{11} & \ldots & t_{1j}^{11} & \ldots & t_{1m_{2}}^{11}\\ \ldots & \ldots & \ldots & \ldots & \ldots \\ t_{i1}^{11} & \ldots & t_{ij}^{11} & \ldots & t_{im_{2}}^{11}\\ \ldots & \ldots & \ldots & \ldots & \ldots \\ t_{m_{1}1}^{11} & \ldots & t_{m_{1}j}^{11} & \ldots & t_{m_{1}m_{2}}^{11} \end{array}\right]. \end{array}$$

Equation (8) is the comprehensive influence matrix *T*_*c*_^*α*^ taking the submatrix *T*_*c*_^11^ as an example after standardization. The sum of the elements in row *i* in *T*_*c*_^11^ is *d*_*i*_^11^. In order to obtain the normalized submatrix *T*_*c*_^*α*11^, divide each element of row *i* in *T*_*c*_^11^ by the row sum of row *i* to obtain the following equation:

(9) $$\begin{array}{c} T_{c}^{\alpha 11}=\left[\begin{array}{ccccc} \frac{t_{11}^{11}}{d_{1}^{11}} & \cdots & \frac{t_{1j}^{11}}{d_{1}^{11}} & \cdots & \frac{t_{1m_{2}}^{11}}{d_{1}^{11}}\\ \ldots & \ldots & \ldots \\ \frac{t_{i1}^{11}}{d_{i}^{12}} & \ldots & \frac{t_{ij}^{11}}{d_{i}^{12}} & \ldots & \frac{t_{im_{2}}^{11}}{d_{i}^{12}}\\ \ldots & \ldots & \ldots \\ \frac{t_{m_{1}1}^{11}}{d_{m_{1}}^{11}} & \ldots & \frac{t_{m_{1}j}^{11}}{d_{m_{1}}^{11}} & \ldots & \frac{t_{m_{1}m_{2}}^{11}}{d_{m_{1}}^{11}} \end{array}\right]\\ =\left[\begin{array}{ccccc} t_{11}^{\alpha 11} & \ldots & t_{1j}^{\alpha 11} & \ldots & t_{1m_{2}}^{\alpha 11}\\ \ldots & \ldots & \ldots \\ t_{i1}^{\alpha 11} & \ldots & t_{ij}^{\alpha 11} & \ldots & t_{im_{2}}^{\alpha 11}\\ \ldots & \ldots & \ldots \\ t_{m_{1}1}^{\alpha 11} & \ldots & t_{m_{1}j}^{\alpha 11} & \ldots & t_{m_{1}m_{2}}^{\alpha 11} \end{array}\right]. \end{array}$$

Then transpose the standardized sub matrix and put it into the corresponding position of the matrix to construct the unweighted supermatrix *W*, set *W*_*ij*_=(*T*_*c*_^*αji*^)′,  *i*=1,2,…, *n*,  *j*=1,2,…, *n*, and standardize it. After calculating the index of index weight, the weighted super matrix *W*_*w*_ is obtained, as shown in the following equation:

(10) $$\begin{array}{c} W_{w}=T_{d}^{\alpha }\times W=\left[\begin{array}{ccccc} t_{11}^{\alpha \;d}\times W_{11} & \ldots & t_{i1}^{\alpha \;d}\times W_{1i} & \ldots & t_{1n}^{\alpha \;d}\times W_{1n}\\ \ldots & \ldots & \ldots \\ t_{i1}^{\alpha \;d}\times W_{i1} & \ldots & t_{ii}^{\alpha \;d}\times W_{ii} & \ldots & t_{in}^{\alpha \;d}\times W_{in}\\ \ldots & \ldots & \ldots \\ t_{n1}^{\alpha \;d}\times W_{in} & \ldots & t_{in}^{\alpha \;d}\times W_{in} & \ldots & t_{in}^{\alpha \;d}\times W_{in} \end{array}\right]. \end{array}$$

Finally, the limit supermatrix is calculated to obtain the authority until the calculation process converges. If the elements of each row of *W*_*w*_ are equal, it indicates that the matrix is in a stable state, and the obtained matrix is the limit supermatrix *W*^*∗*^. Further determine the weight value of the index, as shown in the following equation:

(11) $$\begin{array}{c} W^{*}=\underset{k⟶\infty }{\lim}W_{W}^{k}. \end{array}$$

At the same time, Delphi expert scoring is used to score the direct impact between influencing factors, and then the fuzzy theory is used to fuzzify and defuzzify the scoring, so as to reduce the subjective scoring consciousness of experts, and then a matrix is constructed to reflect the direct scoring, as shown in the following equation:

(12) $$\begin{array}{c} \left\{\begin{array}{c} Y={\left(y_{ij}\right)}_{n\times n},\\ B=\frac{Y}{\underset{1\leq i\leq n}{\max}\left(\sum_{j=1}^{n}y_{ij}\right)}. \end{array}\right. \end{array}$$

In equation (12), *y*_*ij*_ is the expert score after defuzzification; that is, the direct influence value of factor *y*_*i*_ on *y*_*j*_. If *i*=*j* and *y*_*ij*_ are assigned as 0, then each factor has no influence on itself, so the values on the main diagonal of matrix *Y* are all 0, and *B*=(*b*_*ij*_)_*n*×*n*_, (0 < *b*_*ij*_ < 1) is the standardized influence matrix, *T*=*T*(*T*_*ij*_)_*n*×*n*_=*B*+*B*^2^+*B*^3^+⋯⋯+*B*^*n*^, when *n*⟶*∞*, the calculation method of the comprehensive influence matrix *T* is shown in the following equation:

(13) $$\begin{array}{c} T=\sum_{i=1}^{\infty }B^{i}\\ =B{\left(E-B\right)}^{-1}. \end{array}$$

In equation (13), *E* represents the identity matrix and *T* reflects the time relationship of various influencing factors. Then, the affected degree, influence degree, cause degree, and centrality of *T* is calculated, and *D*, *R* represents the row and vector, column and vector of *T*, respectively, as shown in the following equation:

(14) $$\begin{array}{c} \left\{\begin{array}{c} D={\left(D_{i}\right)}_{n\times 1}={\left(\overset{n}{\underset{j=1}{\sum }}C_{ij}\right)}_{n\times 1},\\ R={\left(R_{j}\right)}_{1\times n}^{\prime }={\left(\overset{n}{\underset{i=1}{\sum }}C_{ij}\right)}_{1\times n}^{\prime }. \end{array}\right. \end{array}$$

In equation (14), *D*_*i*_ is the sum of the element values in row *i* of *T*, indicating the influence degree of the element on other elements; *R*_*j*_ is the sum of the *j* column element values of *T*, indicating the influence of this element on other elements. At the same time, the expert opinion coordination coefficient is introduced to promote the optimization evaluation of the model, as shown in the following equation:

(15) $$\begin{array}{c} Vj=\frac{\sqrt{Dj}}{\left(\sum_{i=1}^{mj}Cij/mj\right)}. \end{array}$$

In equation (15), *Vj* is the coefficient of variation, *M*_*j*_ is the number of experts participating in the evaluation of *j* index, *C*_*ij*_ is the scoring system of the *i* expert for the *j* index, and *D*_*j*_ is the mean square deviation of *j* index. The value of the coefficient of variation is inversely proportional to the degree of coordination of experts.

## 4. Application Analysis of Innovative Path in College English MOOCS Teaching

### 4.1. Application Performance Effect Analysis of Innovation Path

**Figure 3:**
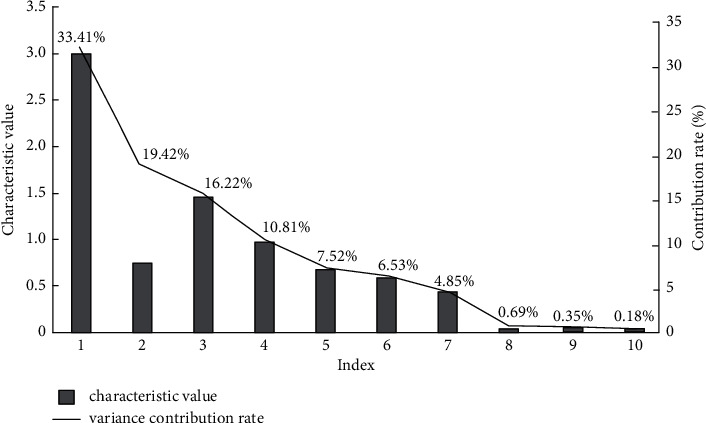
Variance contribution rate of each principal component feature.

The Internet of things collects, monitors, and interacts in real-time with various information-sensing devices to realize the information network between information and the Internet. The integration of a large number of data resources can evaluate the quality of teaching and the effect of its implementation, and then master the teaching feedback of the subject and object of education, so as to provide practical guidance for the exploration of the teaching application path. Starting from the two subjects of students and teachers, the study reconstructs the original teaching quality indicators with the principal component analysis method, selects some data to form the training samples, calculates them according to their weight ratio affecting the teaching quality, counts the variance contribution rate of each principal component feature, and draws a graph. The results are shown in Figure 3.

**Figure 4:**
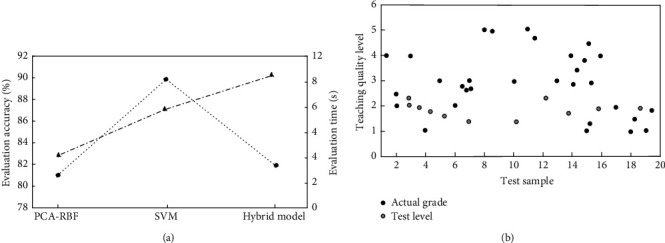
A comparative analysis of the evaluation performance of different evaluation models on English teaching quality: (a) Comparison results of evaluation accuracy and time under different models; (b) Teaching quality evaluation results under mixed model test.

In Figure 3, 1–6 are primary indicators, specifically teaching design, teaching content, teaching effect, teaching innovation, learning effect and learning quality, and the primary indicators are subdivided into secondary and tertiary indicators. It can be seen from the figure that among the eigenvalues of the 10 component features, the component eigenvalues of the teaching content and teaching evaluation feature set are 3.005 and 0.032, and their corresponding contribution rates are 33.392% and 0.182%. The contribution rates of each feature set show a downward trend and fluctuate greatly, and their corresponding eigenvalues also show fluctuations in varying degrees. Compare and analyze the evaluation effect of this algorithm with other algorithms on teaching quality, and the results are shown in Figure 4.

In Figure 4(a), the evaluation accuracy of the principal component analysis radial basis function, PCA-RBF algorithm, and support vector machine (SVM) algorithm on teaching quality is 82.79% and 87.12%, respectively, while the evaluation accuracy of the hybrid model in this paper is more than 90%, which has a good operation effect. The evaluation time is only 3.25 s, which is much faster than the SVM algorithm (10.23 s) and only 0.5 s more than the PCA-RBF algorithm, but its evaluation accuracy exceeds 7.48% of the PCA-RBF algorithm. In Figure 4(b), with the increase of the number of test samples, the evaluation of teaching quality grade by the hybrid algorithm is mostly concentrated above grade 3, and the overall teaching quality is higher than the actual teaching quality grade. On the whole, the hybrid algorithm based on principal component analysis and vector machine performs well in the accuracy and time of evaluating teaching quality, which helps teachers to better improve and improve according to their actual needs.

### 4.2. Performance Analysis of College English MOOCS Innovation Path Application Practice

**Figure 5:**
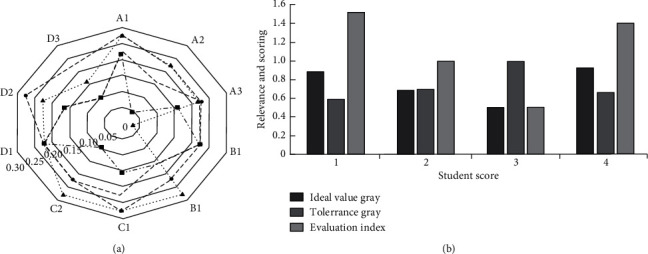
Schematic diagram of evaluation indicators for the English Majors' achievements and their completion. (a) Schematic diagram of standardized scoring of the English major's completion. (b) Evaluation indicators of English major's completion.

By randomly selecting students' scores and learning quality scores under the MOOCS course, an expert scoring table is constructed. The scores are transformed into fuzzy semantics, and on this basis, deblurring is carried out to standardize the scoring table, and the decimal point represents the scoring system of the system. The results are shown in Figure 5.

**Figure 6:**
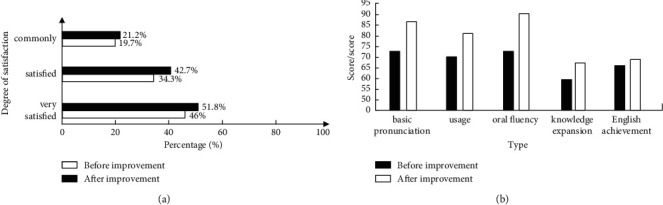
Statistics of students' satisfaction and professional ability scores under the improvement of English teaching path. (a) Statistics of English Major's satisfaction with the course. (b) Statistics of students' professional before and after the improvement of the teaching path.

By randomly selecting students' scores and learning quality scores under the MOOCS course, the expert scoring table is constructed, the scores are transformed into fuzzy semantics, and on this basis, the fuzzy processing is carried out to standardize the scoring table. The results are shown in Figure 5(a). On this basis, the scores of the selected students are sorted, the gray correlation coefficient matrix of the ideal value and tolerance value of the score is further constructed, and finally, the evaluation index score of the score is obtained. The results are shown in Figure 5(b). From the perspective of ideal value and gray value, the fourth student has the highest ideal gray value (0.923) and the lowest tolerance gray value (0.5803); the corresponding scores of the first student are relatively high, the places with relatively low scores are the least, and their comprehensive score is ideal. Overall, the first student (1.1591) and the fourth student (1.4087) have a higher evaluation index. The traditional correlation method only involves the ideal value, which is difficult to comprehensively and effectively evaluate the quality of students' performance. At the same time, a questionnaire was issued to collect students' satisfaction and professional quality improvement after improving the teaching path, and the collected data were plotted. The results are shown in Figure 6.

**Figure 7:**
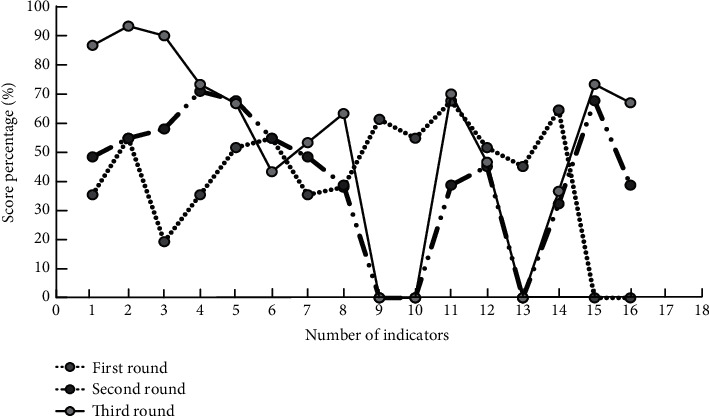
Statistics of satisfaction frequency changes of different indicators after expert review.

In Figure 6(a), the teaching path after innovation and improvement has increased in varying degrees compared with that before improvement, in which the increase of satisfaction has reached 5.8% and 6.4%, and the satisfaction proportion that thinks the effect of the course is general has also reached 21.2% after improvement. In Figure 6(b), English majors have made progress in basic pronunciation, language habits, oral fluency, knowledge development, and professional English scores of 6–25 points. Before improvement, students' professional ability scores are mostly concentrated in the range of 58–73 points. The overall results show that the improved teaching path can effectively meet the professional needs of students. At the same time, the full score frequency of indicators after the expert review is counted, and the results are shown in Figure 7.

In Figure 7, 1–6 refer to the first level indicators, and 7–16 refer to the second-level indicators under the subdivision of the first level indicators. It can be seen from the figure that after three expert reviews, the full score frequency percentage of the first and second level indicators shows an upward trend, and among the first level indicators, the maximum satisfaction frequency of teaching content and teaching effect can reach 96.7% and 91.3%, respectively. After the first and second rounds of expert evaluation, the percentage of Full Score frequency of secondary indicators shows obvious fluctuations. However, after three rounds of evaluation, the overall full score frequency of secondary indicators for evaluating teaching quality gradually increases, and some indicators with full score frequency of 0 are due to their limited evaluation scope and lack of reference objects.

## 5. Conclusion

On the basis of improving the original MOOC teaching path, this study combines the principal component analysis method with support vector machine, uses the network analysis method to fuzzy process and expert review the indicators affecting the teaching path, and constructs and experiments with teaching quality evaluation system. The results show that the highest and lowest component eigenvalues of the feature set are 3.005 and 0.032, respectively, and the corresponding contribution rates of teaching content and teaching evaluation are 33.392% and 0.182%, respectively. The accuracy of the teaching quality evaluation of improved algorithm and hybrid algorithm is at a high level. The evaluation accuracy of the hybrid model proposed in this paper is more than 90%, which has a good operation effect. Its evaluation time is 0.5s more than the PCA-RBF algorithm, and its evaluation accuracy exceeds the PCA-RBF algorithm. At the same time, students' satisfaction and extraordinary satisfaction with the improved teaching path have been improved, respectively. Excellent scores in basic pronunciation, language habits, oral fluency, knowledge development, and English majors. The highest satisfaction with teaching content and teaching effect can reach 96.7% and 91.3%, respectively. In general, the innovation of teaching path can greatly improve students' learning enthusiasm, but the deficiency of this study is that there are few cases selected, and there are still some uncertain factors in the test results we hope to improve in the follow-up study.

## Data Availability

The data used to support the findings of this study are available from the corresponding author upon request.
